# Polyploid evolution in *Oryza officinalis *complex of the genus *Oryza*

**DOI:** 10.1186/1471-2148-9-250

**Published:** 2009-10-14

**Authors:** Baosheng Wang, Zhuoya Ding, Wei Liu, Jin Pan, Changbao Li, Song Ge, Daming Zhang

**Affiliations:** 1 State Key Laboratory of Systematic and Evolutionary Botany, Institute of Botany, the Chinese Academy of Sciences, Beijing 100093, PR China; 2 Graduate University of the Chinese Academy of Sciences, Beijing 100039, PR China

## Abstract

**Background:**

Polyploidization is a prominent process in plant evolution, whereas the mechanism and tempo-spatial process remained poorly understood. *Oryza officinalis *complex, a polyploid complex in the genus *Oryza*, could exemplify the issues not only for it covering a variety of ploidy levels, but also for the pantropical geographic pattern of its polyploids in Asia, Africa, Australia and Americas, in which a pivotal genome, the C-genome, witnessed all the polyploidization process.

**Results:**

Tracing the C-genome evolutionary history in *Oryza officinalis *complex, this study revealed the genomic relationships, polyploid forming and diverging times, and diploidization process, based on phylogeny, molecular-clock analyses and fluorescent *in situ *hybridization using genome-specific probes. Results showed that C-genome split with B-genome at ca. 4.8 Mya, followed by a series of speciation of C-genome diploids (ca. 1.8-0.9 Mya), which then partook in successive polyploidization events, forming CCDD tetraploids in ca. 0.9 Mya, and stepwise forming BBCC tetraploids between ca. 0.3-0.6 Mya. Inter-genomic translocations between B- and C-genomes were identified in BBCC tetraploid, *O. punctata*. Distinct FISH (fluorescent *in situ *hybridization) patterns among three CCDD species were visualized by C-genome-specific probes. B-genome was modified before forming the BBCC tetraploid, *O. malampuzhaensis*.

**Conclusion:**

C-genome, shared by all polyploid species in the complex, had experienced different evolutionary history particularly after polyploidization, e.g., inter-genomic exchange in BBCC and genomic invasion in CCDD tetraploids. It diverged from B-genome at 4.8 Mya, then participated in the tetraploid formation spanning from 0.9 to 0.3 Mya, and spread into tropics of the disjunct continents by transcontinentally long-distance dispersal, instead of vicariance, as proposed by this study, given that the continental splitting was much earlier than the C-genome species radiation. We also find reliable evidence indicated that an extinct BB diploid species in Asia was presumptively the direct genomic donor of their sympatric tetraploids.

## Background

Polyploidization is a prominent process in the evolution of high plants. Between 50% and 70% of angiosperm species were identified as polyploids by intensive screening, while recent studies estimated that up to 100% of angiosperms underwent genome duplication at least once in their evolutionary history [1,2]. The commonity of polyploidy suggests a potential advantage of polyploids to survive better in harsh environments than diploids [3,4]. Thronged facts related to polyploidy were discovered, e.g., genomic divergence in allopolyploids by diploidization, rapid genomic changes, and inter-genomic invasion [5-9]. However, more evidence is needed to reveal the mechanism and tempo-spatial process of polyploidization. Polyploid complex, a group of species with a variety of ploidy levels, could be an ideal model to address the problems. *Oryza officinalis *complex is an excellent example, not only because it contains diploids and heterochronically formed polyploids, but also it has a "pivotal genome" [10], the C-genome, which participated in all the polyploid formation, potentially as an inner criterion to trace polyploid evolution. Moreover, geographic patterns of the polyploids distributed pantropically to isolated continents within a rather recent period, have remained mysterious [11-15].

With agricultural importance, the genus *Oryza *comprises 23 species including cultivated rice, combined into four species complexes [16-19]. In the last decades, molecular methods have been used to reconstruct species phylogeny and to trace evolution process in *Oryza *[14,15,20-26]. Ten distinctive genomes were identified on the basis of DNA sequences [14], or genomic *in situ *hybridization (GISH) [27-29]. Particularly in *O. officinalis *complex, the largest in *Oryza*, genomic relationships were found extraordinarily complicated, e.g., the BBCC tetraploid species formed independently with different parenthood by three polyploidization events, in which *O. eichingeri *was the maternal parent of tetraploid *O. punctata *while diploid *O. punctata *was that of tetraploids *O. malampuzhaensis *and *O. minuta *[14,22,30,31]. Furthermore, three tetraploid species with CCDD genomes were assumed to be formed by one polyploidization event, where the CC genome progenitor served as the maternal parent [14,21,22,32,33]. Additionally, it seems much intriguing that the C-genome diploids and tetraploids are distributed across Asian, African and American tropics. And the CCDD tetraploids are entirely endemic to Central and South Americas where no extant diploid with C- or D-genome was found [11,12]. Therefore, the questions arose: (a) How was the C-genome, as the pivotal genome in all the tetraploids, differentiated after polyploidizating? (b) When were the tetraploids formed and how did they spread transcontinentally? (c) Whether inter-genomic interaction, e.g., exchange or invasion, happened in the allopolyploids?

Focused on the questions, we reconstructed the phylogenetic relationship, dated the divergence time among the genomes in *O. officinalis *complex, and detected genomic changes thereafter polyploidization by FISH methods with genome-specific probes. The goal of this study is to reveal the evolution history of the *O. officinalis *complex, particularly the polyploidization and its genomic impact, by tracing C-genome differentiating and dispersing process.

## Methods

### Plant materials

Thirty eight accessions representing eleven species of *O. officinalis *complex were sampled, and one accession of *O. granulata*, a species outside the complex, was used as outgroup (Table 1). Of them, eight species with different ploidy levels and geographic origins were used for cytogenetic analysis. All the accessions used in this study, are showed in Table 1, including their species names, genome constitutions, original collection locations and GenBank accession numbers. Total DNAs were extracted from fresh leaves of individual plants by the CTAB method [34].

**Table 1:** Plant materials used and all sequences obtained in this study

**Taxa**	**Code^a^**	**Genome**	**Acccession** **No**.^b^	**Origin**	**GenBank Accession No**.	
					
					***Os125***	***SDBE***
*O. officinalis*	off_THA	CC	100179	Thailand	FJ918688	FJ918761
*O. officinalis*	off_VIE	CC	101399	Vietnam	FJ918689	FJ918762
*O. officinalis*^*cd*^	off_BAN	CC	102460	Bangladesh	FJ918690	FJ918763
*O. officinalis*	off_CHI	CC	104618	China	FJ918691	FJ918764
*O. officinalis*	off_MAL	CC	104672	Malaysia	FJ918692	FJ918765
*O. officinalis*	off_IND	CC	104708	India	FJ918693	FJ918766
*O. officinalis*	off_MYA	CC	106390	Myanmar	FJ918694, 695^e^	FJ918767, 768^e^
*O. officinalis*	off_PNG	CC	106519	Papua New Guinea	FJ918696	FJ918769
*O. eichingeri*	eic_UGA1	CC	105159	Uganda	FJ918702	FJ918770
*O. eichingeri*^*c*^	eic_UGA2	CC	105162	Uganda	FJ918701, 703^e^	FJ918771
*O. eichingeri*	eic_LAK1	CC	105407	Sri Lanka	FJ918700, 704^e^	FJ918772, 773^e^
*O. eichingeri*^*c*^	eic_LAK2	CC	104608	Sri Lanka	FJ918705	FJ918774
*O. eichingeri*^*d*^	eic_LAK3	CC	105415	Sri Lanka	FJ918706	FJ918775
*O. rhizomatis*	rhi_LAK1	CC	105440	Sri Lanka	FJ918697, 698^e^	FJ918776
*O. rhizomatis*^*c*^	rhi _LAK2	CC	103414	Sri Lanka	FJ918699	FJ91877, 878^e^
*O. punctata*^*c*^	pun _KEN	BBCC	104975	Kenya	FJ918707, 724^e^	FJ918780, 795^e^
*O. punctata*	pun _UGA	BBCC	105160	Uganda	FJ918708, 722^e^	FJ918781, 794^e^
*O. punctata*^*d*^	pun _IND	BBCC	100125	India	FJ918709, 723^e^	FJ918779, 796^e^
*O. malampuzhaensis*	mal _IND1	BBCC	80765	India	FJ918710, 729^e^	FJ918786, 799^e^
*O. malampuzhaensis*	mal _IND2	BBCC	80767	India	FJ918711, 728^e^	FJ918785, 800^e^
*O. malampuzhaensis*^*cd*^	mal _IND3	BBCC	80768	India	FJ918712, 727^e^	FJ918784, 801^e^
*O. minuta*^*c*^	min _PHI1	BBCC	101141	Philippine	FJ918713, 725^e^	FJ918782, 798^e^
*O. minuta*	min _PHI2	BBCC	104674	Philippine	FJ918714, 726^e^	FJ918783, 797^e^
*O. alta*^*cd*^	alt _SUR	CCDD	100967	Suriname	FJ918715, 731, 739^e^	FJ918787, 802^e^
*O. alta*	alt _BRA	CCDD	100161	Brazil	FJ918755, 756, 757^e^	FJ918812, 817^e^
*O. alta*	alt _GUY	CCDD	105143	Guyana	FJ918752, 753, 754^e^	FJ918810, 816^e^
*O. grandiglumis*^*cd*^	gla _BRA1	CCDD	105669	Brazil	FJ918716, 730, 738^e^	FJ918788, 803, 804^e^
*O. grandiglumis*	gla _BRA2	CCDD	101405	Brazil	FJ918743, 744, 745^e^	FJ918815, 820^e^
*O. grandiglumis*	gla _BRA3	CCDD	105664	Brazil	FJ918758, 759, 760^e^	FJ918814, 821^e^
*O. latifolia*	lat _CRA	CCDD	100167	Costa Rica	FJ918717, 734, 736^e^	FJ918791, 805^e^
*O. latifolia*	lat _PAN	CCDD	100966	Panama	FJ918718, 732, 735^e^	FJ918789, 806^e^
*O. latifolia*^*cd*^	lat _NIG	CCDD	102481	Nigaragua	FJ918719, 733, 737^e^	FJ918790, 807^e^
*O. latifolia*	lat _GUA	CCDD	100171	Guatemala	FJ918749, 750, 751^e^	FJ918811, 819^e^
*O. latifolia*	lat _MEX	CCDD	100914	Mexico	FJ918746, 747, 748^e^	FJ918813, 818^e^
*O. punctata*^*c*^	pun _CAM	BB	105984	Cameroon	FJ918720	FJ918792
*O. punctata*^*d*^	pun _CHA	BB	105607	Chad	FJ918721	FJ918793
*O. australiensis*^*c*^	aus _AUS1	EE	105277	Australia	FJ918740	FJ918808
*O. australiensis*	aus _AUS2	EE	101410	Australia	FJ918741	FJ918809
*O. granulate*^*c*^	gra _LAK	GG	100880	Sri Lanka	FJ918742	FJ918822

^a ^The first three letters represent species name of an accession, followed by its origin country.

^b ^All accessions were provided by the Genetic Resources Center of the International Rice Research Institute (IRRI), Los Banos, the Philippines.

^c ^Accession selected to represent the species for divergence time analyses.

^d ^Accession selected for fluorescent *in situ *hybridization.

^e ^Heterozygous locus with more than one allele.

### Primer design, PCR amplification and sequencing

Two genes, *Starch debranching enzyme *(*SDBE*) on chromosome 4 and *Os02 g0125000 *(*Os125*) on chromosome 2 of *O. sativa*, were chosen in the present study. *SDBE *is a single copy gene [35], containing 25 introns, in which the seventh was used in this study. *Os125 *is also identified as a single copy gene by the criterion previously reported [23], which had three introns and the second one was selected. Primers used for PCR amplifying and sequencing are listed in Table 2.

**Table 2:** Primers used for *SDBE *and *Os125 *gene amplifying and sequencing

**Gene name**	**Primer**	**Sequence(5'-3')**
*Starch Debranching Enzyme*	*SDBE*-F	ATTGTCTGCTGCTGGCTTGA
	*SDBE*-R	CTATTGCCGCTTGTTGCTC
	*SDBE*-Fs2	AAAGGGCAAGCCAACGCAAAT
	*SDBE*-Fs3	TGGACAGCCGACAGACTTGC
*Os02 g0125000*	*Os125*-F	CCAGAAGAATGGGACAGC
	*Os125*-R	GACAGGGAGTTCCAGAGC

Amplification and purification of the PCR products were performed by standard methods. Purified PCR products were sequenced directly or after cloning into pGEM-T-easy vectors (Promega, Madison, WI, USA). Sequencing was performed by ABI 3730 automated sequencer (Applied Biosystems, Foster City, CA, USA). All sequences obtained in this study have been deposited to the GenBank database under accession numbers FJ918688-FJ918822 (Table 1).

### Date analysis

Sequences were aligned with CLUSTAL_X version 1.81 [36]. GC content, base frequency, pairwise divergence and the percentage of phylogenetically informative characters were calculated by MEGA4 [37].

Phylogenetic tree was built using maximum parsimony (MP) and Bayesian inference (BI) methods. MP analyses were performed using heuristic search with 1000 replicates of random stepwise addition and tree bisection-reconnection (TBR) branch swapping in PAUP version 4.0b10 [38]. Gaps were treated as missing data. Bootstrap resampling [39] was conducted to assess topological robustness with 1000 replicates. BI analyses were performed in MrBayes version 3.1.2 [40] by Metropolis-coupled Markov Chain Monte Carlo algorithm. Sequences of each gene were divided into three different partitions (exon, intron and insertion), and the combined data have six partitions. GTR+I+G model was applied for the exon of *Os125*, GTR+G model for the insertion of *SDBE*, and HKY model for the rest. Four Markov chains were conducted for 1,000,000 generations, trees were sampled every 100 generations, and then the first 2500 trees were discarded in the burn-in period. Optimal models and parameters under the Akaike Information Criterion (AIC) were determined by Modeltest 3.06 [41] for Bayesian analyses. When different alleles from heterozygotes were grouped into one clade, one of them was excluded randomly in phylogeny of the combined data, unless they were otherwise grouped into different clades. Congruence between *SDBE *and *Os125 *was evaluated using the partition homogeneity test (PHT) [42], implemented in PAUP with 1000 replicates, random taxon addition (10 replicates), and one tree saved per replicate. Results from the PHT indicated that incongruence between these two genes was P = 0.01, ten folds higher than the suggested (P < 0.001) by Cunningham [43].

Divergence times were estimated by Bayesian dating methods [44-46], using the programs *Baseml *[47], *Estbranches *[44] and *Multidivtime *[46]. Splitting times of *O. officinalis *complex from its affiliated genus *Oryza *and tribe Oryzeae were determined through the plastid gene *matK *of 11 representatives and two outgroups (Table 3). A recent report suggested that origin of Oryzeae was about 34.5 ± 6.8 Mya [48], based on newly discovered pollen fossils [49,50] and phytoliths [51]. These dates were used as the maximum and the minimum constraints to the crown node of Oryzeae, respectively. Other settings were F84+G model [52] and 100,000 MCMC (markov chain monte carlo) iterations, with *rttm *and *rttmsd *set at 6.0, *rtrate *and *rtratesd *set at 0.02, *brownmean *and *brownsd *set at 0.16, and big time set at 100.

**Table 3:** Taxa used for divergence-time analyses in Oryzeae

**Taxon**	**GenBank** **Accession No**.	**Taxon**	**GenBank** **Accession No**.
*O. sativa*	AF148650	*Leersia tisserantii*	AF489901
*O. punctata*	AF148611	*Luziola fluitans*	AY792567
*O. officinalis*	AF148658	*Prosphytochloa prehensis*	AF489916
*O. eichingeri*	AY318858	*Zizania latifolia*	AY092064
*O. australiensis*	AF148667	Ehrharta erecta*	AY792568
*O. granulata*	AF148674	Phyllostachys aurea*	AF1643901
*Leersia oryzoides*	AY792566		

* Outgroups used in the divergence-time analyses in Oryzeae.

For divergence time estimation within the *O. officinalis *complex, an MP tree with 19 sequences representing the taxa of the complex (Table 1) was applied. Insert sequences were excluded due to their considerably variable lengths. Calculations were performed using the same Bayesian relaxed clock methods stated above. Dating constraints between the complex and its outgroup, and between the first clades split within the complex, were set as 13.6 ± 3.6 Mya, and 8.0 ± 2.9 Mya respectively, which were determined by the dating of Oryzeae as described above. Other specific parameters were set as follows: *rttm *and *rttmsd *set to 1.36, *rtrate *and *rtratesd *to 0.04, *brownmean *and *brownsd *to 0.7, according to preliminary dating analysis.

### Preparation of genome specific sequences

C-genome-specific sequences (against B-genome) were isolated by a modified subtractive hybridization methods [53] as follows: genomic DNAs from *O. officinalis *(CC, Accession 102460) and *O. punctata *(BB, Accession 105607) were digested with *MseI *(New England Biolabs, Beverly, MA, USA) into 500 to 1000 bp fragments; and then the fragments of C-genome were ligated with adapter-C and those of B genome were ligated with biotinylated adapter-B (Table 4). Ligation efficiency was checked by PCR amplification using adapter specific primers, C-adp1 and B-adp1. The C-genome ligation was denatured and annealed together with excess B-genome ligation in a single tube. The anneal temperature was 68°C with 0.99 M sodium salt overnight, and then the supernatant containing C-genome-specific sequences was selectively recovered from the reaction mix with streptavidin-coated magnetic beads (Dynabeads, Dynal, Lake Success, NY, USA). A more round of subtracting process was necessary to enrich the genome-specific sequences. Finally, molecules containing the genome-specific sequences were amplified with C-adp1 as primer, and then used for the plasmid transformation.

**Table 4:** Adapters and Primers used in C genome specific sequence preparation

**sequence name**	**primer name**	**Sequence(5'-3')**
CS-1	CS1-F	TTTCCCAATCAAGTTCCT
	CS1-R	ACGGTGGTAATGGTAGCC
CS-2	CS2-F	AAACAGCAGCGGAAAGAG
	CS2-R	GCAAATAGCCATAAGCC
CS-3	CS3-F	CAAACCCAAACCACCCAAGC
	CS3-R	GAACCATACCATCGCCGTCA
CS-4	CS4-F	CTGGTGCCTGCTTTAGTC
	CS4-R	CCATACCGTTGCCTCTTA
CS-5	CS5-F	ACGACCAAGCCGACCAAC
	CS5-R	TGCCTCTTCCACCACTAACT
CS-6	CS6-F	GCTTTGGGTTGGACTTGAC
	CS6-R	TGAACTCGGTGAGATTGGA
CS-7	CS7-F	GGCTGACTGAAGGGAGGAGG
	CS7-R	TGAGGTTGGACGCTGGACTG
CS-8	CS8-F	ATCATTCATTGCTCCATTC
	CS8-R	AACAGCGTCCTCACCAG
adapter-C	C-adp1	GACCTCGTGTCTGCGTACC
	C-adp2	TAGGTACGCAGACACGAG
adapter-B	B-adp1*	GACGATGAGTCCTGAG
	B-adp2	TACTCAGGACTCAT

* 5' end biotinylated

The transformed plasmids were sequenced, and the sequences were BLAST searched in GenBank. Then a series of primers (Table 4) designed from the sequences were used to test whether the sequences were genome-specific or not by PCR amplification onto the related accessions, no correspond bands were seen in BB diploid species (date not shown). Further, the genome-specific sequences were labelled as probes and finally verified by fluorescent *in situ *hybridization (FISH). The FISH images showed no signal on the chromosomes and nuclei of BB diploid species (data not showed). All these data confirmed that the sequences were C-genome-specific.

### Cytogenetics analysis

Chromosome spreads were prepared by enzymatic maceration/air-dry method [54,55]. Total genomic, genome-specific and 45S rDNA probes were labelled by nick translation with biotin-16-dUTP (Roche Diagnostics GmbH, Mannheim, Germany) or DIG-11-dUTP (Roche Diagnostics GmbH, Mannheim, Germany), respectively. Multicolor fluorescence *in situ *hybridization (FISH) was performed as described [29] with slight modification. After overnight hybridization, the slides were given a stringent wash in 20% (v/v) formamide in 0.1× SSC at 42°C, resulting in 80%-85% stringency. The biotinylated-probes were detected by avidin-FITC (Roche Diagnostics GmbH, Mannheim, Germany), and the digoxigenin-labelled probes by anti-digoxigenin rhodamine conjugate (Roche Diagnostics GmbH, Mannheim, Germany). The chromosome spreads were mounted in Vectashield mounting medium with DAPI (Vector Laboratories, Burlingame, CA, USA), and examined under a Leica DMRBE microscope (Leica, Wetzlar, Germany). Photographs were captured by a SPOT cooled color digital camera system (Diagnostic instruments Inc., MI, USA), then imported into Adobe Photoshop 7.0 (Adobe Systems Inc., San Jose, CA, USA) for processing.

## Results

### Sequence characterization

**Figure 1:**
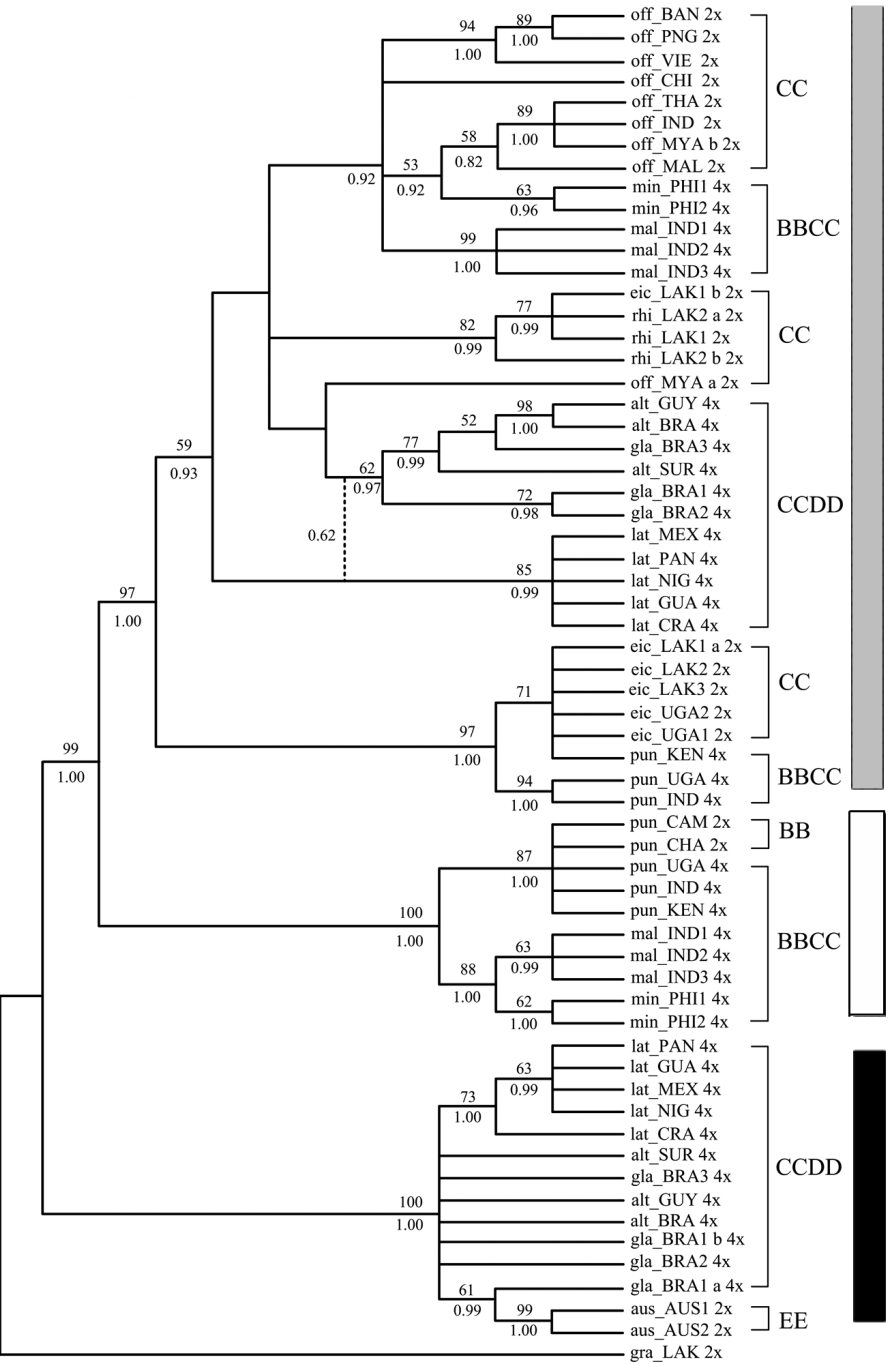
**Strict consensus trees of *SDBE *gene from 72 most parsimonious trees**. Numbers above branches: bootstrap values (only those > 50% showed), below: Posterior probability (only those > 0.5 showed). a or b: alleles of a heterozygous locus. 2× or 4×: ploidy levels. Dash lines indicated the nodes supported by Bayesian inference. Tree length = 184, Consistency index (CI) = 0.8859, Retention index (RI) = 0.9694, Bayesian inference -ln L = -4517.46 (Gray Square: C and C-like copy; White Square: B and B-like copy; Black Square: E and E-like copy).

**Figure 2:**
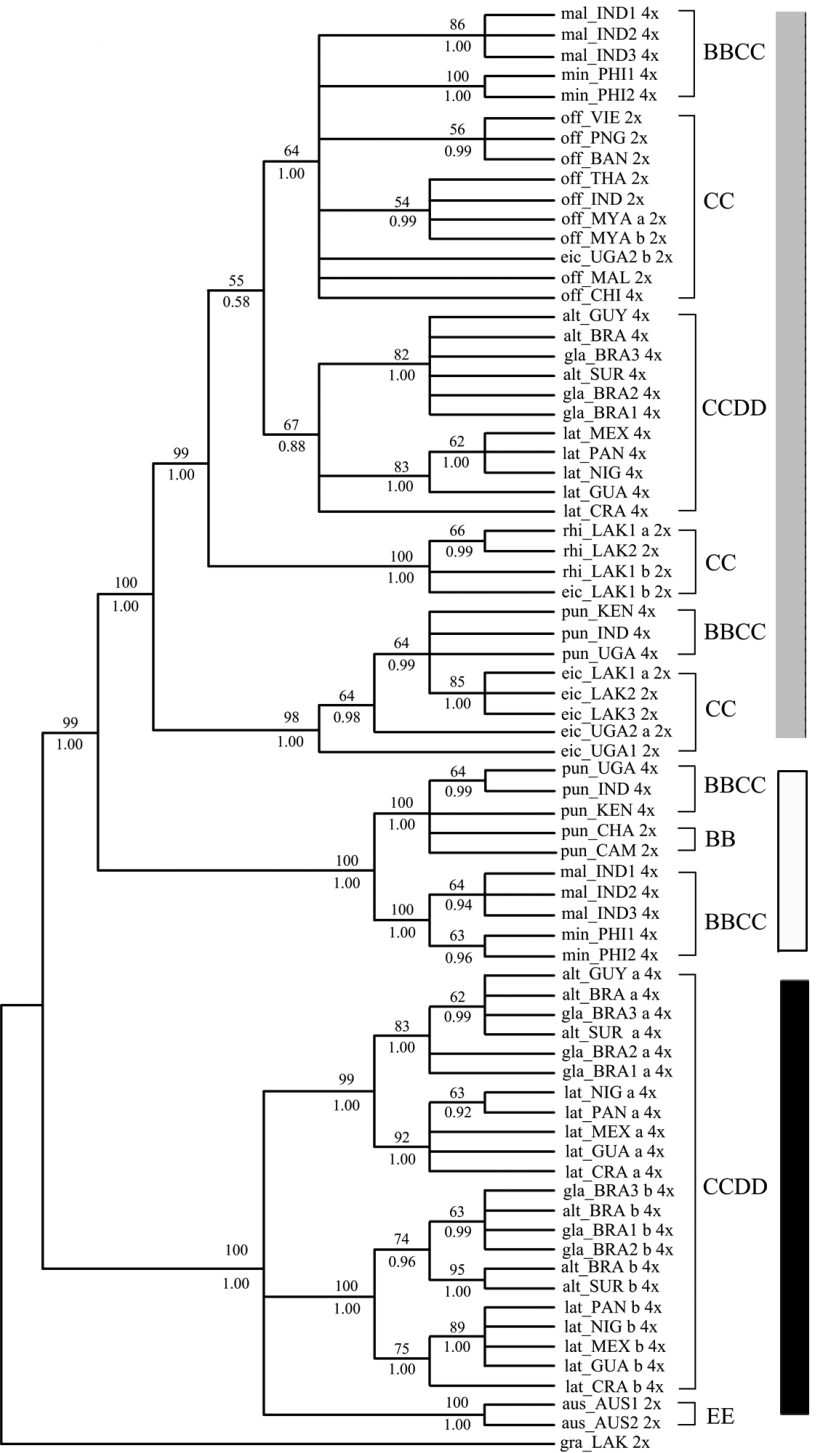
**Strict consensus trees of *Os125 *genes from 500 most parsimonious trees**. Numbers above branches: bootstrap values (only those > 50% showed), below: Posterior probability (only those > 0.5 showed). a or b: alleles of a heterozygous locus. 2× or 4×: ploidy levels. Tree length = 319, Consistency index (CI) = 0.8715, Retention index (RI) = 0.9807, Bayesian inference -ln L = -3585.67 (Gray Square: C and C-like copy; White Square: B and B-like copy; Black Square: E and E-like copy).

**Figure 3:**
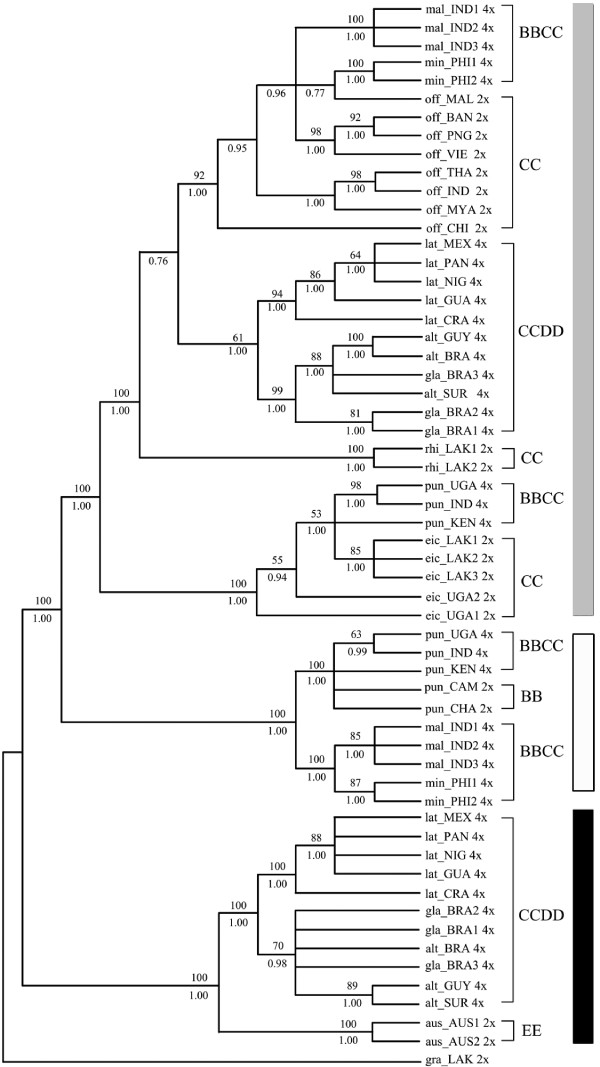
**Strict consensus trees of combined data set from 126 most parsimonious trees**. Numbers above branches: bootstrap values (only those > 50% showed), below: Posterior probability (only those > 0.5 showed). a or b: alleles of a heterozygous locus. 2× or 4×: ploidy levels. Tree length = 463, Consistency index (CI) = 0.8985, Retention index (RI) = 0.9771, Bayesian inference -ln L = -7735.44 (Gray Square: C and C-like copy; White Square: B and B-like copy; Black Square: E and E-like copy).

Two distinct sequences in both *SDBE *and *Os125 *genes were identified from each accession of all tetraploids. One was longer (740-2272 bp in *SDBE *and 871-1021 bp in *Os125*) and the other was shorter (420-458 bp in *SDBE *and 400-701 bp in *Os125*). Each longer sequence was highly similar to and phylogenetically grouped with the corresponding sequences of CC diploids (Figures 1, 2, 3), and thereby was named as C-like copy. Each shorter one was similar to that of BB or EE diploids, and thus was named as B- or E- like copy accordingly. Aligned sequence of *SDBE *(2337 bp) had 101 (4.3%) informative sites, and that of the *Os125 *(1112 bp) contained 164 (14.7%) informative sites. The combined sequence of these two genes was aligned to be 3449 bp in length (Table 5).

**Table 5:** Characteristics of each gene and combined dataset

**Locus**	**Aligned length (range)**	**GC (%)**	**Mean sequence divergence (range)(%)**	**Variable sites (%)**	**Informative sites (%)**
*SDBE*	2337(420-2272)	34.7	4.2 (0-11.6)	155 (6.6)	101 (4.3)
*Os125*	1112(400-1021)	38.6	7.1 (0-20.6)	256 (23.0)	164 (14.7)
combined	3449(1112-3290)	36.8	5.6 (0-15.7)	389 (11.3)	249 (7.2)

Sequence divergence was estimated using the Jukes-Cantor distance

A ~320 bp insertion in *SDBE *and a ~150 bp insertion in *Os125 *were recognized in each of the C-genome-bearing species. BLAST searches in the TIGR rice repeated database  and then mask against Repbase Update  using CENSOR [56], identified the insertion of *SDBE *to be MITE-*MDM2 *(miniature inverted transposable element-*MDM2*), but no matching sequence of the *Os125 *insertion was found. The *Os125 *insertion was flanked by a short direct repeat (sequence: TACATGGCTCTTTC), but no terminal inverted-repeating sequence nor tRNA-related region was found, suggesting that this fragment is an unidentified retrotransposable element instead of a SINE (short interspersed repetitive element) [57].

In addition, a partial fragment (~1.5 kb) of L1-type retrotransposon family was found to insert into C-like *SDBE *gene in some accessions of *O. alta *and *O. grandiglumis*.

### Phylogeny reconstruction based on *SDBE*, *Os125 *and combineddataset

Phylogenetic analyses of *SDBE*, *Os125 *and combined dataset using maximum parsimony (MP) and Bayesian inference (BI), all yielded similar topologies. Parsimony analysis yielded 72, 500 and 126 equally most parsimonious trees, from *SDBE, Os125 *and combined dataset, respectively. The strict consensus trees of each dataset were showed in Figures 1, 2 and 3 with general features as follows: (a) The main clades were strongly supported by bootstrap values and Bayesian posterior probability. (b) B-, C- and E-like copies in tetraploid species formed three monophyletic clades with the corresponding sequences of BB, CC and EE diploid species, respectively. (c) In the C-genome clade, two monophyletic clades were formed, one involving *O. eichingeri *and the tetraploid *O. punctata*, and the other covering all the rest C-genome species. (d) The B-like copies of BBCC tetraploid species were divided into two clades, one including the Africa endemic *O. punctata *(comprising BB diploid and BBCC tetraploid), and the other including the Asian tetraploids only. We also put the *SDBE *and *Os125 *sequences of *O. sativa *(A-genome, GenBank Accession No. AB012915 and AP004885) into the datasets, but the positions of B-, C- and E-like copies, and the topology of the inferred cladegram, remained unchanged (data not showed).

With more informative sites, the cladograms of the complex constructed from *Os125 *and combined dataset were more resolvable (Figures 2, 3). *O. officinalis *(CC) and C-like copies of the BBCC tetraploids, i.e., *O. malampuzhaensis *and *O. minuta*, were consistently united into one clade. The clade was further grouped with C-like copies of all CCDD tetraploid species. Apart from the C-like copies of *Os125 *sequences, two E-like copies were isolated from CCDD species, which formed two clades in parallel and finally grouped with *O. australiensis *(EE) trichotomously in both MP and BI trees (Figure 2).

It is noteworthy that two alleles of the heterozygous accessions were grouped with each other, except eic_LAK1 and eic_UGA2, in which one of the alleles was clustered with that of different species (Figures 1, 2), suggesting that interspecific hybridization and introgression in those accessions occurred, as proposed by previous research [58]. For those heterozygous loci, the allele that clustered into the *O. eichingeri *clade, was selected in the combined dataset.

### Divergence Dates

**Figure 4:**
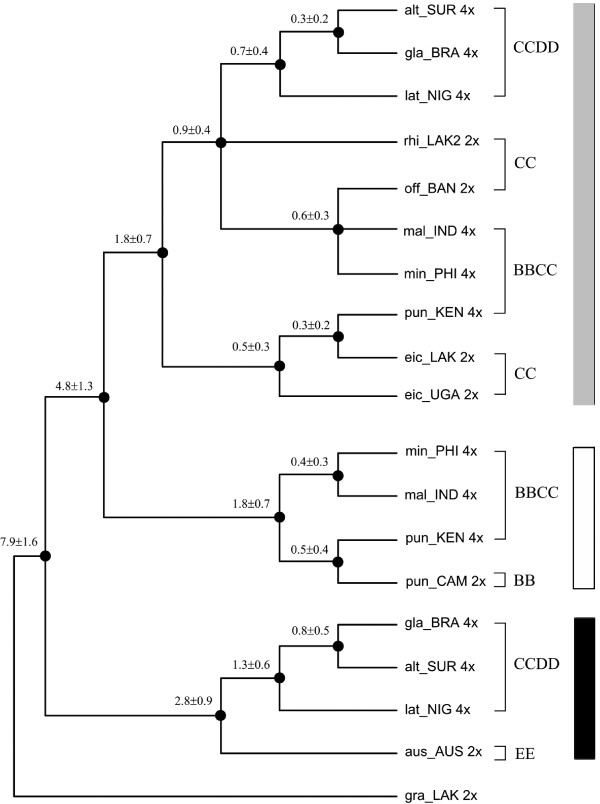
**Divergence times of main lineages in *O. officinalis *complex**. Calculated by Bayesian relaxed-clock methods (details see Materials and Methods). Estimated Mya and the standard deviation were noted above the branches. 2×: diploid; 4×: tetraploid (Gray Square: C and C-like copy; White Square: B and B-like copy; Black Square: E and E-like copy).

As showed in Figure 4, the *O. officinalis *complex was estimated to diverge from the rest of the genus *Oryza *at 7.9 ± 1.6 Mya, and the separation between B- and C- genomes took place at 4.8 ± 1.3 Mya. The molecular dating indicated that three C-genome diploid species radiated between ca. 0.9-1.8 Mya during Pliocene. In BBCC tetraploid species, C-like copy of *O. punctata *diverged from C genome of *O. eichingeri *at 0.3 Mya, very close to the divergent time (0.5 Mya) of the B-like copy from the B genome of diploid *O. punctata*. In other two BBCC species (*O. malampuzhaensis *and *O. minuta*), the C-like copies diverged from their common paternal progenitor (*O. officinalis *alike) at ca. 0.6 Mya, later than the divergence time of their B-like copies from the B-genome of *O. punctata *(BB) at ca. 1.8 Mya. Similarly, the divergence time between C-like copies of CCDD tetraploids and their C-genome donor, was set at ca. 0.9 Mya, while the node to separate their D-genomes from *O. australiensis *(EE) was dated at ca. 2.8 Mya (Figure 4).

### FISH analysis

**Figure 5:**
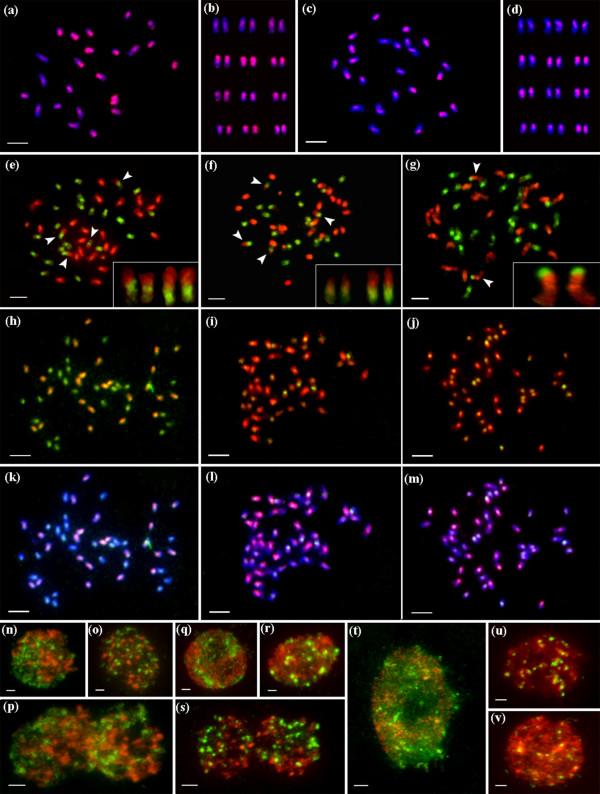
**Multicolor fluorescent *in situ *hybridization images of *O. officinalis *complex**. Hybridized by C genome-specific probes (red), together with B genome probes (green, e-g, n-s) or E genome probes (green, h-m, t-v), counterstained by DAPI (blue, a-d, k-m). Arrows indicated inter-genomic (B-C genomes) translocations, which were enlarged in the below box (e-g). (a) *O. officinalis *(CC), and its karyotype (b). (c) *O. eichingeri *(CC), and its karyotype (d). (e-f) *O. punctata *(BBCC). (g) *O. malampuzhaensis *(BBCC). (h, k) *O. latifolia *(CCDD). (i, l) *O. grandiglumis *(CCDD). (j, m) *O. alta *(CCDD). Parental genomes in the nuclei of allotetraploids, separated spatially in *O. punctata *(n, interphase; o, prophase; p, anaphase), in *O. malampuzhaensis *(q and r, interphase stages; s, anaphase), and in interphase nuclei of *O. latifolia *(t), *O. grandiglumis *(u), and *O. alta *(v), respectively. Bar, 5 μm.

Figure 5 shows multicolor fluorescent *in situ *hybridization images of *O. officinalis *complex, hybridized by C-genome-specific probes (red), together with B-genome probes (green) or E-genome probes (green), counterstained by DAPI (blue).

Figures 5a-d show that the C-genome-specific probes were localized on all chromosomes of two diploid CC species, *O. officinalis *and *O. eichingeri*. The C- genome-specific sequences were scattered non-uniformly along each of the chromosome as well as among twelve homologous pairs, as the FISH patterns showed obviously (Figures 5a, c). The total 24 chromosomes were karyotypically arranged into twelve homologous pairs according to their FISH patterns, relative length, centromere position and heterochromatin, as showed in Figure 5b and Figure 5d.

Figures 5e and 5f show the FISH images of the tetraploid *O. punctata *using digoxigenin-labelled C-genome-specific probes and biotin-labelled total genomic DNA of diploid *O. punctata *(BB). B- and C-genomes were clearly discriminated in the same nucleus, where 24 chromosomes showed strong bright green signals of the B-genome probes, and the rest 24 chromosomes showed strong red signals of the C-genome-specific probes (Figures 5e, f). It is worth to notice that two pairs of B-genome chromosomes were clearly involved in inter-genomic translocations with the C-genome, one small and the other rather large.

Figure 5g shows that a prometaphase nucleus of *O. malampuzhaensis *was hybridized with C- genome-specific probes (red) together with B-genome probes (green). The 24 B-genome chromosomes exhibited strong green signals, and the rest 24 chromosomes belonging to C-genome showed bright red signals. Two B-genome signals were identified at short arm terminals of one pair of C-genome chromosomes. However, when *O. malampuzhaensis *was hybridized with B-genome probes (green) together with 45S rDNA probes (red), these two C-genome chromosomes with 45S rDNA signals were also painted by B-genome signals on same areas (Additional file 1). Therefore, in *O. malampuzhaensis *which was with different origin from tetraploid *O. punctata*, B-genome signals located on the two C-genome chromosomes may not be inter-genomic translocation but homologous sequences of 45S rDNAs.

Multicolor FISH was also used for three CCDD species, where two probes were applied, one from *O. australiensis *(EE) genome (labelled in green), and one from the C-genome-specific probe (labelled in red). Figure 5h shows in *O. latifolia*, strong C-genome-specific signals (orange) painted 24 chromosomes, while green signals (from the E-genome probes) stained all chromosomes, in which 24 chromosomes with pure green signals should belong to the D-genome. The FISH patterns of *O. latifolia *differed remarkably from those of *O. alta *and *O. grandiglumis*. In *O. latifolia *all chromosomes were painted by E-genome signals (Figures 5h, K), whereas in *O. alta *and *O. grandiglumis *all chromosomes were painted by C-genome signals (red), in which merely some of the chromosomes showed the E-genome signals (green) faintly or strongly near centromere regions (Figures 5i-j; 5l-m). This difference could also be seen in the interphase cells, as showed in Figures 5t-v, where nuclei of *O. latifolia *were dominantly painted by E-genome probes while those of *O. alta *and *O. grandiglumis *were strongly painted by C-genome-specific probes with dot-like signals of E-genome probes.

Figure 5n-s shows each of the two parental genomes separated spatially in BBCC tetraploid species in interphase, prophase and anaphase nuclei. In *O. malampuzhaensis*, about 10 chromocenters of B-genome were found at late stage of interphase (Figure 5r); however, no similar chromocenters were found in *O. punctata*.

## Discussion

The key to trace the complicated evolution process of polyploid complex lies on a universal criterion. C-genome in *O. officinalis *complex could play such a role. As the pivotal genome, C-genome participated each of the polyploid formation in the complex, and its evolution process in genomic differentiation and geographical patterning can therefore reflect the temporal and spatial history of polyploid evolution.

### Genomic relationships in *O. officinalis *complex

In *O. officinalis *complex, four extant genomes, B, C, D or E, were identified [14,21,27]. The present study showed that each genome in the complex occurred only once when rooted by the outgroup, *O. granulata*, where E-genome sited at the basal position of the complex. The clade of *O. officinalis *complex was first divided into two clades, E-genome clade and the other clade involving B-and C-genomes. In E-genome clade, D-genome was located as E's sister group. These results were consistent with previous reports [14,21-23]. In the other clade, C-genomes in different diploid species had differentiated apparently thereafter they partook in different polyploid formation (Figures 1, 2, 3), in agreement with other authors [58-61].

Although there is only one extant diploid with B-genome, *O. punctata*, the B-genomes in tetraploids were differentiated, as revealed by AFLP [21], RFLP [62], SSR [60] and GISH [29]. In this study, multicolor FISH (Figure 5g) revealed that the B-genome of *O. punctata *(BB) was clearly diverged from that of *O. malampuzhaensis*. Further evidence of molecular phylogeny and dating showed that the divergence happened even before polyploidization, which formed *O. malampuzhaensis *and *O. minuta *(Figures 1, 2, 3, 4). Therefore, a diploid B-genome species extinct nowadays in Asia was assumed to be the direct genomic donor of Asian distributed BBCC tetraploids.

Since no diploid DD species has ever been found, the D donor for the CCDD tetraploids has long been controversial [14,27,28,32]. The Australian diploid, *O. australiensis*, as the unique E-genome holder, was assumed to be D-genome donor by several authors [14,32]. Nevertheless, genomic comparison by GISH and retrotransposon analysis found obvious differences between D- and E-genomes, and thus suspected E as the direct donor [28,63]. Based on a universal criterion of C-genome differentiation, our study in phylogeny and molecular dating (Figures 1, 2, 3, 4) showed that D- and E-genome were tied together as sister group, but they diverged much earlier than CCDD tetraploid formation. Multicolor FISH using E-genome probes for the CCDD tetraploids also revealed obvious differentiation between D- and E-genome, and this was even remarkable in D-genome itself, as showed in Figure 5, where the D-genomes of *O. alta *and *O. grandiglumis *exhibited sharply different from that of *O. latifolia*.

### C-genome variation and polyploid evolution in *O. officinalis *complex

To date six tetraploid species, three BBCC and three CCDD, have been recorded in *O. officinalis *complex, and all are C-genome carriers [16,64]. The relationship and origin of the tetraploids have long been in debate [14,21,22,32]. In this study, C-genome of diploid *O. eichingeri *was localized at the basal of C-genomes, and it subsequently diverged, resulting two C-genome diploids, *O. rhizomatis *and *O. officinalis*. Later on, the three C diploids participated separately in hybridization and polyploidization, finally forming six tetraploids. For *O. eichingeri*, it merely joined formation of *O. punctata *(BBCC), while *O. officinalis *(CC) partook in formation of *O. malampuzhaensis *and *O. minuta*. On the other hand, a species closely related to present *O. officinalis *(or *O. rhizomatis*) offered its C-genome to the three CCDD tetraploids, *O. alta*, *O. grandiglumis *and *O. latifolia *(Figures 1, 2, 3).

C-genomes in different BBCC tetraploids confronted variable fates, such as changes by inter-genomic translocation. Multicolor FISH probing different genomes in an allopolyploid can be a powerful indicator for identifying such changes. As showed in multicolor FISH (Figure 5e-g), inter-genomic translocations between C-and B-genomes were visualized for the first time in two tetraploids of the complex, which was speculated as the result of diploidization impact [5,8,65,66]. In *O. punctata *two fragments of C-chromosomes were translocated to different B-chromosomes, while in *O. malampuzhaensis *no obvious inter-genomic translocation was found. Although C-genomes experienced different history in various polyploid formations, few fragments of C-genome-specific were detectably lost after hybridization and polyploidization, as found in multicolor FISH with genome-specific probes (Figures 5e-g, n-s).

The fate of C-genomes in CCDD tetraploids was different even more. In *O. alta *and *O. grandiglumis *C-genome-specific probes apparently dominated the nuclei, most probably by inter-genomic invasion [5,8,67,68], as showed in multicolor FISH images, while in *O. latifolia *C-genome kept almost unchanged (Figures 5 5h-m, t-v). Considering that *O. alta *and *O. grandiglumis *diverged from *O. latifolia *(Figure 4), the inter-genomic invasion would have happened during their speciation.

### Temporal and spatial evolution of *O. officinalis *complex

Geographical pattern of intercontinental pantropics in *O. officinalis *complex, framed by its relatively recent history, makes its evolution process paradoxical for long time. Based on molecular clock of *matK *and *GAP1 *sequences, the origin of the complex was dated at late Miocene (ca.9 Mya) [69], and speciation of *O. australiensis *was set in ca. 8.5 Mya through *Adh2 *gene [63]. However, re-dating the origin and divergence times became necessary, because (a) previous dating dealt mainly with diploids while the polyploids evolution history remained unclear; (b) new molecular timescales based on non-parametric rate smoothing, penalized likelihood, and Bayesian-relaxed clock methods have been recently developed for the grasses [70].

In this study, the estimated divergence time between *O. officinalis *complex and its outgroup, *O. granulata*, was 13.6 ± 3.6 Mya, earlier than the previous suggestion, and the time of the first species divergence in the complex, was 7.9 ± 1.6 Mya (Figure 4). C-genome was separated with B-genome at about 4.8 Mya, and then C-genome itself was split into two clades in approximately 1.8-0.9 Mya, one including *O. eichingeri *and the other including the rest two CC diploid species. These times were earlier than previously suggested [58], but closed to recent research [24,25]. The time of polyploidization to synthesize tetraploids was estimated to be ca. 0.3-0.9 Mya in Pleistocene, in which the CCDD species (ca. 0.7-0.9 Mya) were formed obviously earlier than BBCC species, also closed to that recently reported [24].

**Figure 6:**
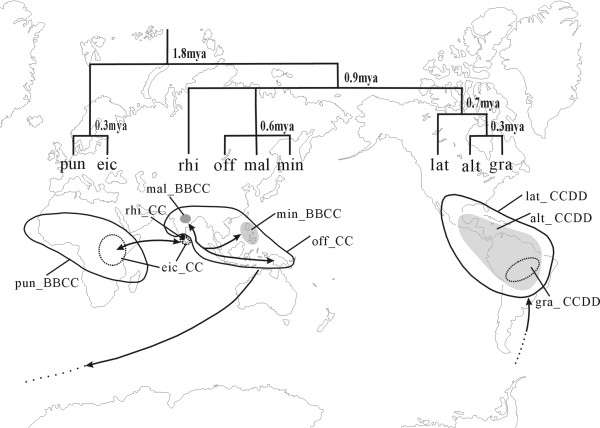
**Biogeographical scenario of species with C genome in *O. officinalis *complex**. Above: divergence time calculated using Bayesian relaxed-clock methods (the cladegram narrowed from Figure 2). Below: Distribution areas (outline and noted) and inferred migration procedure among continents. CC diploids (eic = *O. eichingeri*, off = *O. officinalis*, rhi = *O. rhizomatis*), BBCC tetraploids (pun = *O. punctata*, mal = *O. malampuzhaensis*, min = *O. minuta*), and CCDD tetraploids (lat = *O. latifolia*, alt = *O. alta*, gra = *O. grandiglumis*).

If all C-genome species separated no earlier than two Mya, the distribution of these species can be feasibly explained by long-distance dispersal rather than vicariance, given that the continental splitting was much earlier than the species radiation. As suggest by Vaughan et al [11,12], animal migration may play a role for this complex in seed dispersal between Asia and Africa. Bird could be another carrier, which could account for the disjunctive distribution of some *Oryza *species, such as *O. eichingeri *[11,58]. For the CCDD tetraploids, this and previous studies [32] both revealed that their putative parents were *O. officinalis *and *O. australiensis*. The problem was that the putative parents were confined to south Asia-Australia but the CCDD tetraploids were nowadays endemic to the tropics of Americas. Therefore, a new pathway to bridge these two continents for long-distance dispersal was put forward (Figure 6). The strong floristic affinities between the South America and the antipodes were also confirmed by biogeographical studies of other Poaceae species [70,71]. However, how the species in the complex could transcontinentally spread across the oceans, remains mysterious.

## Conclusion

The genomic relationships, polyploid formations and divergence times in *Oryza officinalis *complex of the genus *Oryza*, were revealed based on DNA sequences and FISH evidence. Focused on C-genome, the "pivotal genome" of the polyploids, we found that the polyploids were formed by stepwise polyploidizations in ca. 0.3-0.9 Mya, followed by a series of inter-genomic translocations and invasions. The pantropical distribution of the complex was suggested to be formed by long-distance dispersal transcontinentally, instead of vicariance. This study offers a typical example in tracing tempo-spatial process of polyploidization, and for the first time it gives new stands for the complex in dating the detailed times of polyploid formation, visualizing inter-genomic changes, and viewing the spatial evolution history of the polyploids.

## Authors' contributions

BW carried out the molecular and cytogenetic studies, wrote the manuscript and participated in the design of the study. ZD, WL and JP made equal contributions in chromosome preparation, data analyses, and phylogenetic inference. CL provided partial DNA sequences for phylogenetic analysis. SG identified all the *Oryza *materials and modified the manuscript. DZ contributed to the design of the study, supervised the experiment steps, and prepared the manuscript. All authors read and approved the final manuscript.

## Supplementary Material

Additional file 1 **Multicolour fluorescent *in situ *hybridization images of *O. malampuzhaensis***. Prometaphase chromosomes of *O. malampuzhaensis *were hybridized by 45S rDNA probes (red) together with B-genome probes (green), and counterstained by DAPI (blue). Arrow indicated one pair of C-genome chromosomes which painted by both 45S rDNA and B-genome signals on same areas. (a) and (d) Two pairs of 45S rDNA loci (red). (b) and (e) The B-genome chromosomes showing blue-green signals. (c) and (f) The chromosomes counterstained by DAPI after hybridized with both 45S rDNA and B-genome probes. Bar, 5 μm. Click here for file
