# Supplementary figures and images for: Polyploid evolution in Oryza officinalis complex of the genus Oryza
Source: BMC Evol Biol. 2009 Oct 14;9:250. doi: 10.1186/1471-2148-9-250 (PMC2770061; doi:10.1186/1471-2148-9-250)

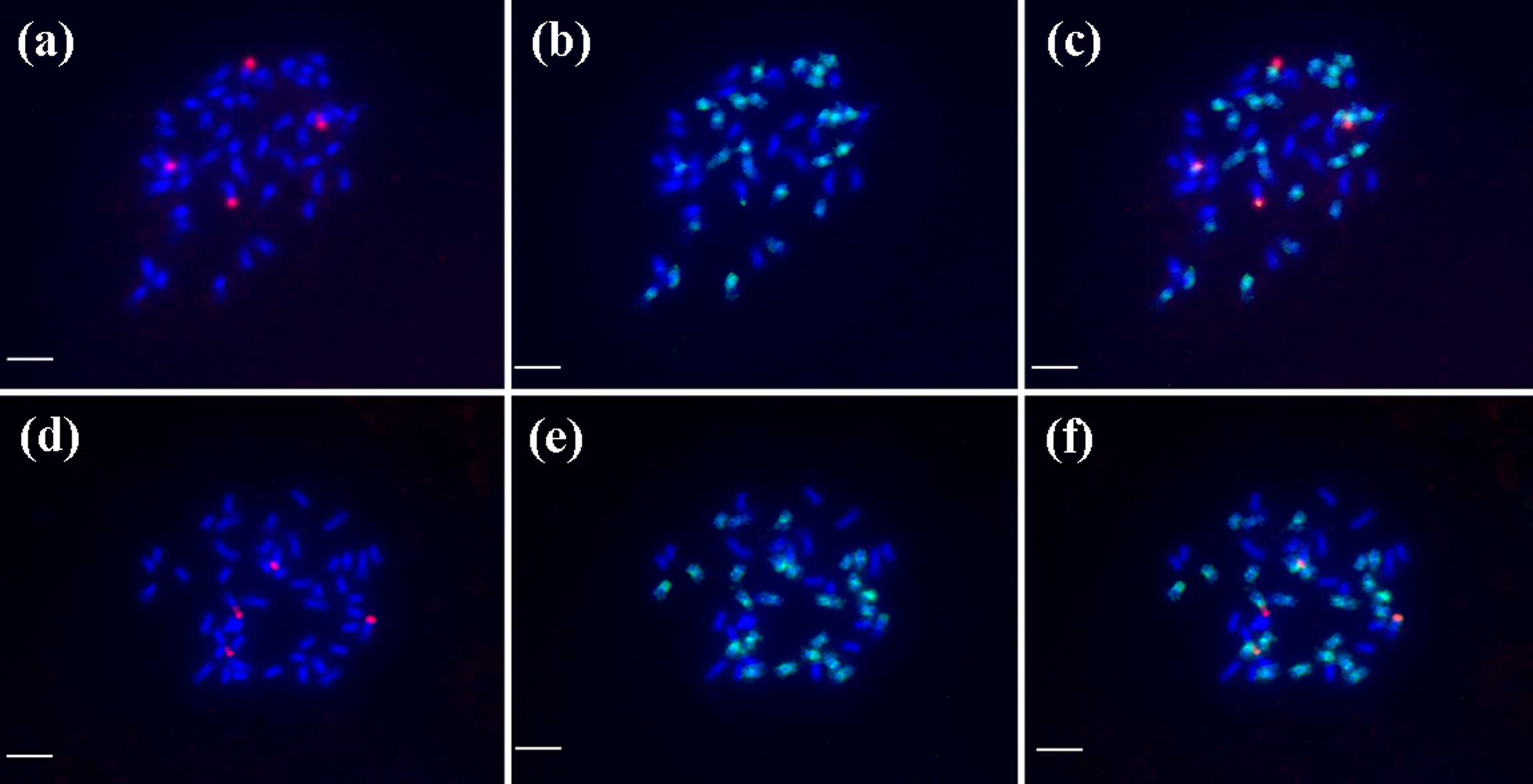

Supplement: Additional file 1 — Multicolour fluorescent in situ hybridization images of O. malampuzhaensis. Prometaphase chromosomes of O. malampuzhaensis were hybridized by 45S rDNA probes (red) together with B-genome probes (green), and counterstained by DAPI (blue). Arrow indicated one pair of C-genome chromosomes which painted by both 45S rDNA and B-genome signals on same areas. (a) and (d) Two pairs of 45S rDNA loci (red). (b) and (e) The B-genome chromosomes showing blue-green signals. (c) and (f) The chromosomes counterstained by DAPI after hybridized with both 45S rDNA and B-genome probes. Bar, 5 μm. [file 1471-2148-9-250-S1.TIFF]
